# Effective Removal of Glyphosate from Aqueous Systems Using Synthesized PEG-Coated Calcium Peroxide Nanoparticles: Kinetics Study, H_2_O_2_ Release Performance and Degradation Pathways

**DOI:** 10.3390/polym15030775

**Published:** 2023-02-03

**Authors:** Fan Li, Thomas Shean Yaw Choong, Luqman Chuah Abdullah, Siti Nurul Ain Md. Jamil, Nurul Nazihah Amerhaider Nuar

**Affiliations:** 1 Department of Chemical and Environmental Engineering, Faculty of Engineering, Universiti Putra Malaysia, Serdang 43400, Selangor, Malaysia; 2 Institute of Tropical Forestry and Forest Product (INTROP), Universiti Putra Malaysia, Serdang 43400, Selangor, Malaysia; 3 Department of Chemistry, Faculty of Science, Universiti Putra Malaysia, Serdang 43400, Selangor, Malaysia; 4 Centre of Foundation Studies for Agricultural Science, Universiti Putra Malaysia, Serdang 43400, Selangor, Malaysia

**Keywords:** calcium peroxide, nanoparticles, AOPs, kinetics study, H_2_O_2_ release, agricultural wastewater

## Abstract

Glyphosate (N-phosphonomethyl glycine) is a non-selective, broad-spectrum organophosphate herbicide. Its omnipresent application with large quantity has made glyphosate as a problematic contaminant in water. Therefore, an effective technology is urgently required to remove glyphosate and its metabolites from water. In this study, calcium peroxide nanoparticles (nCPs) were functioned as an oxidant to produce sufficient hydroxyl free radicals (·OH) with the presence of Fe^2+^ as a catalyst using a Fenton-based system. The nCPs with small particle size (40.88 nm) and high surface area (28.09 m^2^/g) were successfully synthesized via a co-precipitation method. The synthesized nCPs were characterized using transform infrared spectroscopy (FTIR), X-ray diffractometry (XRD), Brunauer–Emmett–Teller analysis (BET), dynamic light scattering (DLS), and field emission scanning electron microscopy (FESEM) techniques. Under the given conditions (pH = 3.0, initial nCPs dosage = 0.2 g, Ca^2+^/Fe^2+^ molar ratio = 6, the initial glyphosate concentration = 50 mg/L, RT), 99.60% total phosphorus (TP) removal and 75.10% chemical oxygen demand (COD) removal were achieved within 75 min. The degradation process fitted with the Behnajady–Modirshahla–Ghanbery (BMG) kinetics model. The H_2_O_2_ release performance and proposed degradation pathways were also reported. The results demonstrated that calcium peroxide nanoparticles are an efficient oxidant for glyphosate removal from aqueous systems.

## 1. Introduction

**Figure 1:**
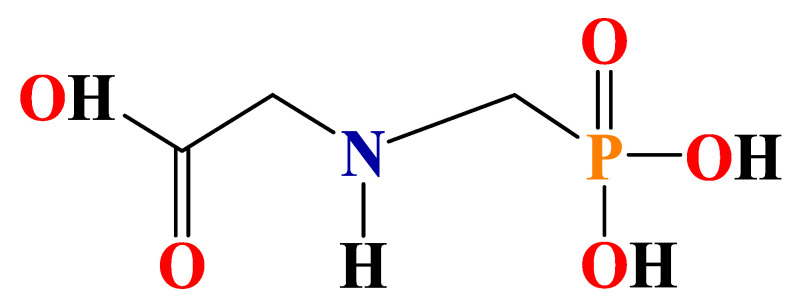
Chemical structure of glyphosate.

Water pollution caused by herbicides is one of major global concerns due to the potential negative effects of these compounds on ecosystems and human health (e.g., cardiac and respiratory impacts) [1]. Particularly, glyphosate containing wastewater has drawn considerable attention to public. Glyphosate, known as PMG or Roundup (commercial formulation), is globally used as an organophosphorus post-emergence herbicide to manage the development of weeds, with an annual production volume of ~700,000 tons [2]. Its widespread application in agriculture in massive quantities could bring about environmental issues, considering the biosafety concerns [3]. Hence, it can be widely detected in surface water (originated from agricultural fields) and municipal wastewater [4]. For instance, the concentration of glyphosate in surface or groundwater was found to be in the range of 2–430 μg/L in the USA [5], which is higher than that in Europe: 0.59–165 μg/L [6,7]. Owing to the three polar functional groups (-PO_3_H_2_, -COOH and -NH-) in molecular glyphosate (shown in Figure 1), glyphosate has proven to be a more challenging water contaminant to effectively remove [8]. Thus, there is an urgent need to develop an effective method to remove glyphosate and its metabolites, such as aminomethylphosphonic acid (AMPA) and sarcosine, from the aqueous environment.

Several methods have been employed to treat wastewater contaminated with glyphosate, mainly including adsorption, biological oxidation, reverse osmosis, and advanced oxidation processes (AOPs) [9]. Among these, physicochemical processes (adsorption, coagulation, and reverse osmosis) are non-destructive, and post-treatments of adsorbent materials or solid wastes are required and costly [10,11]. Biological processes (bacteria and fungi) are eco-friendly but limited to lab scale. Besides, another drawback of the above methods is that the metabolites (e.g., AMPA, sarcosine) with higher toxicity cannot be entirely eliminated [12]. In the past years, AOPs (photolysis-based, Fenton-based, electrochemical and ozonation oxidation) have acquired widespread acceptance as potential effective methods for treating glyphosate containing wastewater [13]. AOPs typically operate at or near ambient temperature and pressure conditions involving the production of powerful oxidizing agents in sufficient quantity for water purification. The main advantages of AOPs are attributed to the shorter treatment time, higher removal efficiency (>90%), less limitation, and a better capacity to remove intractable compounds or metabolites. Fenton-based oxidation is preferentially utilized among AOPs for wastewater treatment [14]. Due to the high standard potentials (2.8 V vs. NHE) and oxidation capacity in acidic media, the hydroxyl radical (·OH) is significantly crucial in AOPs [15]. The large amount of free hydroxyl radicals (·OH) are generated from the reaction between Fenton reagents, which are H_2_O_2_ and Fe^2+^, and the reaction equation is shown in the Equation (1).

H_2_O_2_ + Fe^2+^ →·OH + OH^−^ + Fe^3+^ (1)

Calcium peroxide (CaO_2_, CP) is considered as a liquid form of H_2_O_2_ to generate ·OH with higher stability and environmental friendliness [16]. In fact, CaO_2_ can dissolve in water to form H_2_O_2_ in acidic condition through Equation (3), liberating a maximum of 0.47 g H_2_O_2_/g CaO_2_ [17]. However, in alkaline condition, CaO_2_ is directly converted to molecular oxygen instead of H_2_O_2_ as described in the Equation (2).

CaO_2_ + H_2_O → 0.5O_2_ + Ca(OH)_2_ (2)

CaO_2_ + 2H_2_O → H_2_O_2_ + Ca(OH)_2_ (3)

Assorted studies have assessed the efficacy of calcium peroxide on pollutants removal. For instance, the removal of fluticasone propionate and clobetasol propionate [18], trichloroethylene (TCE) [19,20], methylene blue [21], and 2,4-dichlorophenol [22] by CP has been reported. In the recent years, nanoparticles CaO_2_ (nCPs) have acquired the researcher’s attentions due to the noteworthy and preferable performance than the commercial CP [16,17]. Results from previous research suggested that the reaction rate of synthesized nCPs was more rapid than commercial CP with silver nanoparticles [23]. Besides, the surface stabilizers used during the synthesis process, such as polyethylene glycol (PEG), polyvinyl pyrrolidone (PVP), polyvinyl alcohol (PVA), dextran, and diethylene glycol monomethyl ether (DEGMME), also dominated the removal efficiency [24]. These surface stabilizers adsorb on nanoparticles and prevent irreversible agglomeration, resulting in stable dispersion with higher steric repulsion [19]. The previous studies have confirmed that nCPs preformed effectively in the removal of organic micropollutants and inorganic heavy metals [25,26,27], acid blue 161 [28], acrylic acid [29,30], benzene [31], and doxycycline [14]. Therefore, the excellent properties of CaO_2_ to generate ·OH at a controlled rate drew an increasing number of researchers to apply CaO_2_ in wastewater industry.

To this end, the objectives of the present study were to synthesize nCPs and to evaluate its performance on glyphosate removal from aqueous systems. The synthesis process was based on a co-precipitation technique, using calcium chloride (CaCl_2_) as a precursor and polyethylene glycol 200 (PEG 200) as a surface stabilizer. The synthesize nCPs were characterized by transform infrared spectroscopy (FTIR), X-ray diffractometry (XRD), Brunauer–Emmett–Teller analysis (BET), dynamic light scattering (DLS), potassium permanganate titration method, and field emission scanning electron microscopy (FESEM) techniques. The removal process of glyphosate from aqueous systems was investigated, utilizing nCPs as an effective oxidant in Fenton-based system. The fitting of different kinetics models was conducted, and the H_2_O_2_ release as well as proposed degradation pathways were also assessed.

## 2. Materials and Methods

### 2.1. Chemical Reagents

Roundup (commercial grade, 41%, containing 360 g/L glyphosate) was diluted to prepare a 1000 mg/L glyphosate stock solution with a natural pH of 4.9–5.9. Calcium peroxide (CaO_2_, 65%), ammonium molybdate (H_8_MoN_2_O_4_, 99%), and ascorbic acid (C_6_H_8_O_6_, 99%) was purchased from Alfa Aesar, Ward Hill, MA, USA. Calcium chloride (CaCl_2_, ≥99.5%), hydrogen peroxide (H_2_O_2_, 30%), ammonia solution (NH_4_OH, 25 wt%), ferrous sulfate (FeSO_4_∙7H_2_O, ≥98%), and ethyl alcohol (C_2_H_5_OH, 95%) were supplied from R&M Chemicals Sdn. Bhd. (Semenyih, Malaysia). Polyethylene glycol 200 [HO(C_2_H_4_O)nH, PEG 200], antimony potassium tartrate [K_2_Sb_2_(C_4_H_2_O_6_)_2_, 99%], sodium sulfite (Na_2_SO_3_, ≥98%, 2.0 M), sodium hydroxide (NaOH), and sulfuric acid (H_2_SO_4_, 95%) were procured from Sigma Aldrich (St Louis, MO, USA). Cerium (IV) sulfate tetrahydrate (CeO_8_S_2_∙4H_2_O, ≥98%) was purchased from Acros Organics, Geel, Belgium. All the chemical reagents used in this study were of analytical reagent grade. Distilled water was used throughout the experiments to prepare the solutions and the stock solutions were freshly prepared every week. The pH of the solutions was adjusted by the addition of 0.1 M H_2_SO_4_ solution and 0.1 M NaOH solution.

### 2.2. Synthesis of Calcium Peroxide Nanoparticles (nCPs)

In this study, calcium peroxide nanoparticles were synthesized utilizing co-precipitation technique with minor modifications [23]. Initially, 3.0 g of CaCl_2_ was dissolved in the 30 mL distilled water and heated to 50 °C to accomplish complete dissolution. 15 mL NH_4_OH (1 M) and 120 mL of PEG 200 solution were added into the stirring mixture. The PEG 200 solution functioned as a steric stabilizer and inhibitor of irreversible agglomeration of nanoparticles throughout the precipitation. After 20 min of agitation, 15 mL of 30% H_2_O_2_ was added to the stirring mixture dropwise with the burette at the rate of 20 drops per minute. The above mixture was constantly stirred for 2 h until a beige to yellowish solution was obtained. The agitation speed throughout the synthesis process was 350 rpm at the ambient temperature. Subsequently, to precipitate the product, 0.1 M NaOH solution was added dropwise until the pH of solution was at 11.50. The appearance of beige to yellowish precipitates indicated the formation of nCPs. The beige to yellowish colored precipitates were separated by centrifugation and after the centrifugation process the powder was washed three times with ethanol. Eventually, two additional washes by distilled water were carried out. The resultant precipitate was dried at 80 °C for 24 h in an evacuated oven to obtain the nCPs. The evacuated oven was used to avoid contamination which may cause impurity in samples. It also permitted the drying process of nCPs in the lower temperature. Equation (4) describes the overall reaction involved in the synthesis process of nCPs.

CaCl_2_∙6H_2_O + 2H_2_O +H_2_O_2_ +2NH_3_ → CaO_2_∙8H_2_O + 2NH_4_Cl (4)

**Figure 2:**
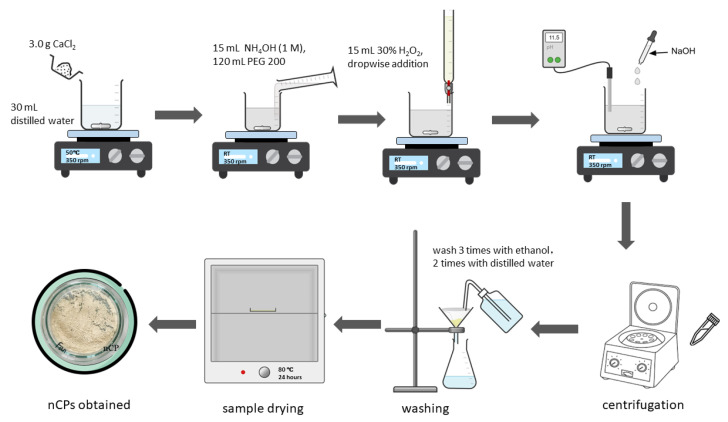
A schematic diagram of synthesis of nCPs.

A schematic diagram for synthesis process of nCPs is displayed in Figure 2.

### 2.3. Fenton-Based Reaction Experiment Procedure

In a typical experiment, the stock glyphosate-containing solution (1000 mg/L) was diluted to 50 mg/L and placed in the 250 mL conical flasks. The equal initial dosage (0.2 g) of nCPs and CP were weighed out and added into the solutions, respectively. The required amount of FeSO_4_ was calculated and added, based on our previous research [13] where Ca^2+^/Fe^2+^ molar ratio was kept at 6. The pH of the solutions was adjusted to 3. The experiments were equipped in a water bath at the agitation speed of 150 rpm, at ambient temperature. The excess of 2.0 M Na_2_SO_3_, as an inhibitor to terminate the Fenton reaction, was added to the samples, assuring the elimination of the remaining H_2_O_2_ in the solutions [32]. The contact time was 2 h, and the absorbance values were monitored at an interval of 15 min. At the first 15 min, sampling was carried out every 5 min. The precipitated supernatant was taken and filtered with 0.22 μm membrane filters before analyses. Two parameters, the total phosphorus (TP) value and chemical oxygen demand (COD) value, were utilized to evaluate the glyphosate removal process. All experiments were conducted in triplicate, and the mean values were reported.

### 2.4. Analytical Method

The pH of the solutions was measured using a digital pH meter (Model: Sartorius PB-10). The ammonium molybdate spectrophotometry method was employed to determine TP values with an ultraviolet-visible spectrophotometer (Dynamica, HALO DB-20). The absorbance of each sample was determined at λ = 880 nm and the calibration curve of standard phosphorus (R^2^ = 0.9980) was obtained. The total removal efficiency was calculated via the Equation (5).

(5) $$\mathrm{Total}\;\mathrm{removal}\;\mathrm{efficiency}\;\left(\%\right)=\left(1-\frac{C_{t}}{C_{0}}\right)\times 100$$

where C_0_ and C_t_ are the concentration of total phosphate at the initial and a given contact time *t*, respectively.

**Figure 3:**
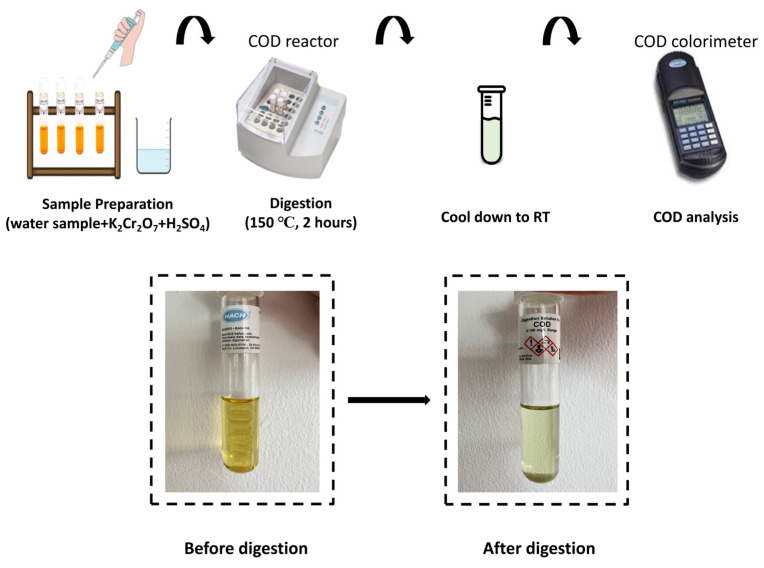
The flowchart of COD determination.

The dichromate digestion-colorimetric method (Lovibond thermoreactor RD125, DR/890 Portable colorimeter) was adopted to analyze the COD values before and after treatment. The total COD removal percentage was calculated using Equation (6), and the flowchart of COD determination is shown in Figure 3.

(6) $$\mathrm{COD}\;\mathrm{removal}\;\left(\%\right)=\left(\frac{{\mathrm{COD}}_{\mathrm{before}\;\mathrm{treatment}}-{\mathrm{COD}}_{\mathrm{after}\;\mathrm{treatment}}}{{\mathrm{COD}}_{\mathrm{before}\;\mathrm{treatment}}}\right)\times 100$$

The potassium permanganate titration method was used to determine the purity of the nCPs sample. The purity was determined using the Equation (7) below.

(7) $$\mathrm{Purity}\;\mathrm{of}\;\mathrm{calcium}\;\mathrm{peroxide}\;\left(\%\right)=\frac{5\times C\left({\mathrm{KMnO}}_{4}\right)\times V\left({\mathrm{KMnO}}_{4}\right)\times 72.08}{2\times m\left({\mathrm{CaO}}_{2}\right)\times 100}$$

where C(KMnO_4_) represents the concentration of standard KMnO_4_ solution, which was 0.02 M in the present study. V(KMnO_4_) indicates the volume (mL) of consumed KMnO_4_ solutions. m(CaO_2_) indicates the mass of sample to be analyzed, which, for each sample in the present study, was 0.05 g. Titration was run in triplicate and the mean value was reported.

The FTIR spectra of CP and nCPs were analyzed via a compact FTIR Spectrometer (Bruker ALPHA II, MA, USA) in the wavelength range of 4000–500 cm^−1^. X-ray diffraction (XRD) analysis was conducted to identify the structure and phase analysis of CP and the synthesized nCPs by X-ray diffractometer (PHILIPS PW 3040/60 MPD X’Pert high Pro-PAN analytical). The software applied to analyze the XRD data was X′Pert HighScore Plus. The surface morphology of the CP and nCPs was characterized by Nova NanoSEM 230 FE-SEM. The particle size distribution from FESEM results were obtained using the software, SPSS. The textural properties of CP and nCPs were acquired by Brunauer–Emmet–Teller (BET) analysis (degassed at 200 °C for 2 h in a nitrogen environment at 77 K) using the Autosorb-1 (Quantachrome Co., Hampshire, UK). The average particle size and polydispersity index (PDI) of the CP and nCPs (dispersant: ethanol) were measured using a nanosizer (Nano S, Malvern Instruments Ltd., Malvern, UK). The release of H_2_O_2_ from CP and nCPs was analyzed via the cerium sulfate spectrophotometry method using a UV spectrophotometer (Dynamica, HALO DB-20). The wavelength used in this study was 480 nm and the calibration curve of Ce(SO_4_)_2_ was prepared and saved for further study (R^2^ = 0.9993). Equation (8) was used to calculate the instantaneous H_2_O_2_ concentration.

(8) $$H_{2}O_{2}\;\mathrm{concentration}\;=\frac{C_{0}V_{0}-C_{t}\left(V_{0}+V_{\mathrm{sample}}\right)\times 0.017}{V_{\mathrm{sample}}}\times 1000$$

where C_0_ and C_t_ represent the initial CeSO_4_ concentration and that of at the given time, *t*, respectively. V_0_ indicates the volume (mL) of the cerium sulfate standard solution and Vsample represents the volume (mL) of the unknown water sample.

### 2.5. Kinetic Study

The reaction kinetics of glyphosate removal by CP and nCPs were examined by pseudo-zero order, pseudo-first order, pseudo-second order, and BMG models. The BMG model was initially proposed by Behnajady, Modirshahla, and Ghanbery in 2007 [33] and was fitted with degradation data in representative research [34,35,36]. The equations are listed in Table 1.

## 3. Results

### 3.1. Characterization

#### 3.1.1. FTIR Spectra of CP and nCPs

**Figure 4:**
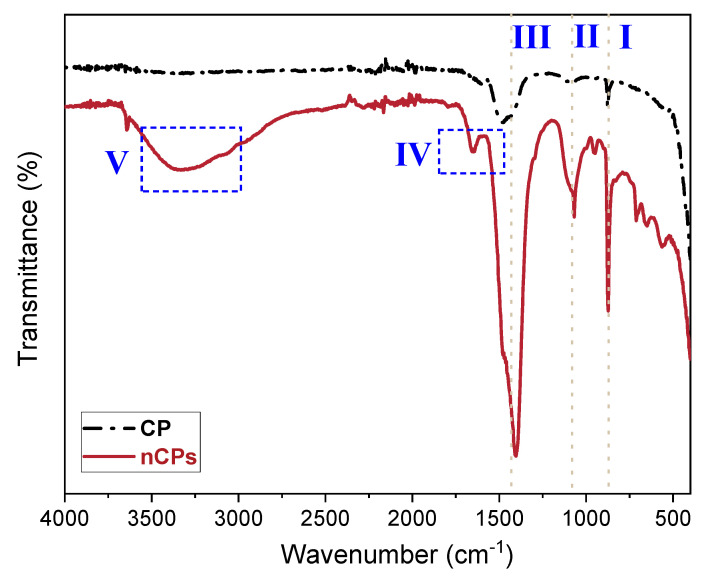
FTIR spectra of CP and nCPs.

The FTIR spectra of calcium peroxide and synthesized nCPs were acquired within the range 4000 to 400 cm^−1^ and presented in Figure 4. The spectra were examined for the functional groups for further confirmation of nCPs. The common and characteristic absorption peaks from two samples were denoted by dashed lines or boxes with numbers I–IV. The common peaks present at 855–880 cm^−1^ (dashed line I) together with the range of 710–750 cm^−1^ corresponded to the O–O bridge of CaO_2_ [37], and that of 520–565 cm^−1^ referred to the O–Ca–O vibrations [19]. An intense peak near 1450–1490 cm^−1^ (dashed line III) was detected for both CP and synthesized nCPs samples, manifesting the O–Ca–O stretching in CaO_2_. A sharp absorption peak near 1100 cm^−1^ (dashed line II) in nCPs sample was identified as the presence of the carbonate ion (CO_3_^2−^) [38], while this signal was weak in CP sample. This finding implied that the synthesized nCPs contained calcite (CaCO_3_) as an impurity. Another weak peak detected near 1660 cm^−1^ (dashed box IV) was attributed to C=C stretch in nCPs sample [38]. Additionally, a broad absorption peak (dashed box V) at the range of 3000–3650 cm^−1^ was identified as the O-H vibration from the surface stabilizer and water molecules [39].

#### 3.1.2. XRD Patterns of CP and nCPs

**Figure 5:**
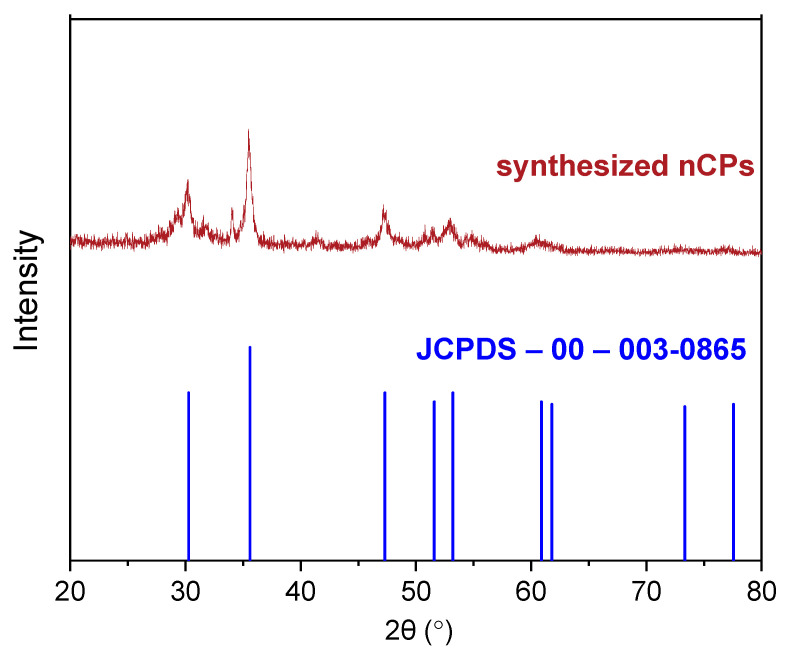
XRD spectra of CP and nCPs.

X-ray diffraction (XRD) analysis of nCPs samples was carried out in the 2θ range from 20° to 80° using a CuKα radiation (λ = 0.1540 nm, 30 mA, 40 kV) to ascertain the chemical composition and calculate the crystallite size. As indicated in Figure 5, the five dominant peaks were detected in synthesized calcium peroxide nanoparticles at 2θ values of 30.18, 35.90, 47.20, 51.90, and 60.94, consistent with standard XRD card data of calcium peroxide (JCPDS-00-003-0865) [40]. In addition, calcite is the sole calcium product precipitated throughout the synthesis other than CP, and no other distinctive peak was detected in the XRD pattern [19]. The primary cause of calcite formation was the carbonation of calcium hydroxide, which is generated because of hydrolysis of the precipitated calcium peroxide during the synthesis [41]. This conclusion accorded with our earlier observations from FTIR results and the purity result from the latter part (Section 3.1.3). Table 2 compares the 2θ and d-spacing values of standard CP with the synthesized nCPs, and the analogy validates its chemical composition. The XRD results firmly confirmed that nCPs was successfully synthesized by this method.

The Debye–Scherrer equation is displayed as Equation (9) and was utilized to calculate the average particle size of nCPs.

(9) $$D=\frac{K\lambda }{\beta cos\theta }$$

where k is the Debye–Scherrer constant (k = 0.9), *λ* is the wavelength of the incident X-ray radiation (*λ* = 0.1540 nm), *θ* is the Bragg’s angle in radians, and *β* is the full width at half maximum (FWHM). The most intense peak at 2*θ* = 35.90 was used to calculate the average particle size. The calculated nanocrystalline size of nCPs was 38.69 nm.

#### 3.1.3. Physicochemical Properties of CP and nCPs

**Figure 6:**
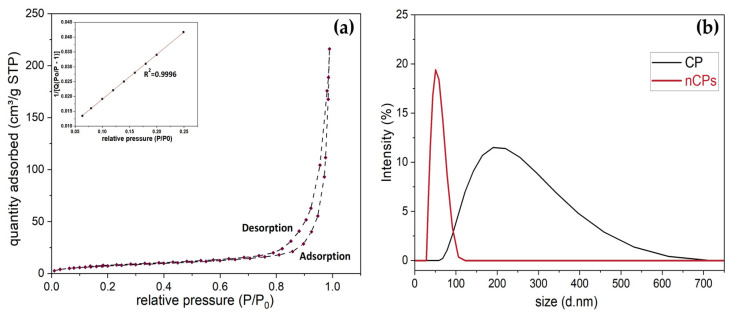
(**a**) BET nitrogen adsorption isotherm plot. (**b**) DLS results of CP and nCPs.

Table 3 presents the physicochemical properties of CP and nCPs, including the results of potassium permanganate titration, BET and DLS analysis. The BET isotherm and the result of DLS analysis are shown in Figure 6. The purity of Ca^2+^ in calcium peroxide samples increased from 65.0% to 75.1%, which reduced the production of byproducts and contaminants. Thus, the main impurity was found to be CaCO_3_ at about 24.90%. Of note, the surface area drastically improved for synthesized CaO_2_ nanoparticles. The surface area of the typically 65% CP was only 3.1079 m^2^/g and of synthesized nCPs was 28.0860 m^2^/g. This remarkable improvement would greatly increase the reaction rate and strengthen the performance of removal. The higher surface area of nCPs corresponded to the larger pore size (23.1324 nm) and larger pore volume (1.3248 cm^3^/g) and the smaller average particle size (40.88 nm) obtained from DLS analysis. Notably, the result of average particle size was relatively in agreement with the XRD result. The PDI value (0.228) indicated that the synthesized PEG-coated nCPs were uniformly dispersed without much agglomeration while the PDI value of CP (0.592) was greatly larger. As shown in Figure 6b, the width of peaks also certified the PDI result. The pore size (23.1324 nm) demonstrated that the synthesized PEG-coated nCPs were well-defined mesoporous (between 2–50 nm). The higher ratio of surface-volume of nCPs samples also confirmed that it would perform better in terms of removing the pollutants. It could be observed from Figure 6a that the limiting gas uptake over high P/P_0_ (0.4–1.0) may be caused by capillary condensation that occurred in pores with monolayer–multilayer adsorption [19]. Likewise, this feature revealed that the adsorption process of nCPs belonged to IUPAC type IV isotherm [42]. Collectively, the enhanced physicochemical properties would be conducive to the greater removal performance.

#### 3.1.4. FESEM Results of CP and nCPs

**Figure 7:**
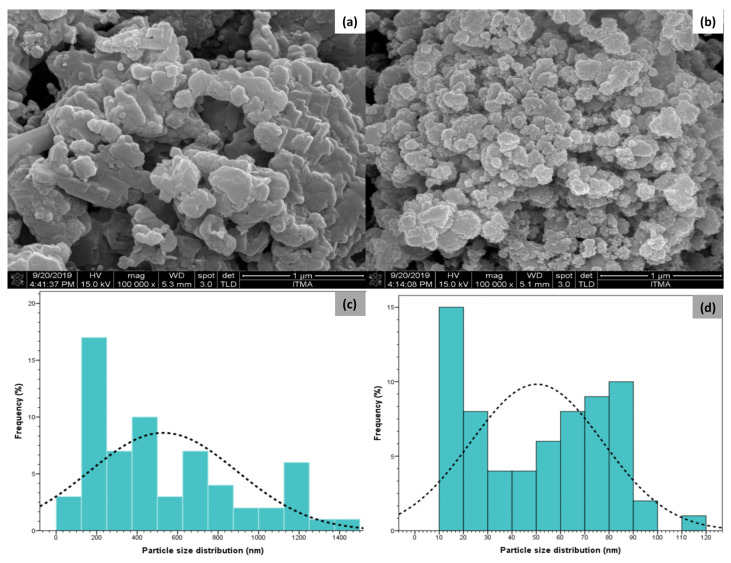
FESEM images of (**a**) CP (**b**) nCPs; Particle size distribution of (**c**) CP (**d**) nCPs.

Figure 7 presents the morphology of samples determined via FESEM with the same magnification (100,000 X) and the particle size distribution of CP and nCPs. Figure 7a shows that conventional CP appeared as irregular flakes of particles in pieces with excessive agglomeration. This would significantly reduce the surface area and removal efficiency. The minimum size was approximately 98.99 nm whereas the maximum size was relatively 1.19 μm due to the exceeding agglomeration. The particle size distribution of CP was mostly between 90 and 400 nm, shown in Figure 7c, as correlated with the DLS results (average particle size: 220.16 nm; PDI: 0.592). In contrast, observed from Figure 7b, the heterogeneous surface morphology with regular mostly spherical shape and smaller particle size could be detected on nCPs, contributing to the higher removal performance. Apparently, the CP nanoparticles were uniformly distributed, and the agglomeration was mitigated. The minimum and maximum particle sizes of nCPs were approximately 10 nm and 94.28 nm, respectively. The particle size distribution of nCPs was mainly at the range of 15–60 nm, illustrated by Figure 7d. Remarkably, PEG 200, which was an effective stabilizer and dispersant to mitigate agglomeration, was attributed to the smaller particle size and better distribution of nCPs [19]. This finding was in great agreement with the previous studies [24,29], which reported the heterogeneous surface morphology of nCPs.

### 3.2. Glyphosate Removal Process Using Fenton-Based System

**Figure 8:**
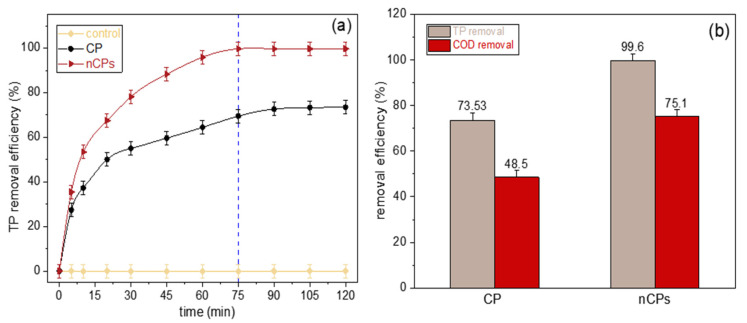
(**a**) The effect of contact time on TP removal based on nCPs using Fenton-based system (Condition: pH, 3.0; the initial dosage of CP and nCPs, 0.2 g; Ca^2+^/Fe^2+^ molar ratio, 6; initial glyphosate concentration, 50 mg/L; RT; 150 rpm). (**b**) Comparison of total removal efficiency of CP and nCPs.

Figure 8 indicates the glyphosate removal process based on CP and nCPs using a Fenton-based system. The optimal experimental conditions obtained from our prior investigation were applied to the present study, with minor modifications [13]. The investigation was carried out at the initial pH = 3.0, the initial calcium peroxide (CP and nCPs, respectively) dosage = 0.2 g, Ca^2+^/Fe^2+^ molar ratio = 6, the initial glyphosate concentration = 50 mg/L under room temperature at the stirring speed of 150 rpm. The smaller particle size with larger surface area and higher purity of calcium peroxide nanoparticles significantly enhanced the removal process. Thus, the higher removal efficiency was achieved compared to the CP. This clearly indicated that the degradation of glyphosate was largely dependent on particle size. It is notable that within 75 min (marked as a vertical dashed line), up to 99.60% removal was reached using nCPs while at the same contact time the removal efficiency using CP was only 69.48%. Within contact time = 75 min, the total phosphorus in the solution was removed effectively by the powerful free hydroxyl radicals (·OH) generated from the Fenton reagents. However, between contact time = 75 min and 120 min, the removal efficiency of CP was still climbing from 69.48% to 73.53% and the equilibrium was detected around 90 min. The reason was that the surface area of conventional CP was quite smaller than nCPs, resulting in the slower removal rate and longer removal time. Additionally, the Fenton reaction involving Fe^2+^ as a catalyst often proceeds in two stages, a fast one and a slower one [35]. The faster stage is caused by an interaction between Fe^2+^ and H_2_O_2_, whereas the slower stage is attributed to the accumulation of Fe^3+^ and the limited recovery of Fe^2+^ via H_2_O_2_ [43]. This behavior was observed in the previous research [39,44] and was also supported by the finding of Figure 8a. The total removal efficiency is displayed in Figure 8b. The TP and COD removal of glyphosate-containing solution using nCPs were considerably more effective compared to the conventional CP. Under the same condition, total 75.10% COD removal was detected while only 48.50% COD removal using conventional CP. Hence, the nCPs is preferred in terms of COD removal because it is an important parameter in the treatment of wastewater with a high phosphorus content. Comparing our total removal efficiencies with the previous studies under the same mechanism and similar experimental conditions [13,45,46], the TP removal efficiency improved by 3.9–5.4% and 6.5–13.0% improvement was detected for COD removal.

### 3.3. Effect of Initial Glyphosate Concentration

**Figure 9:**
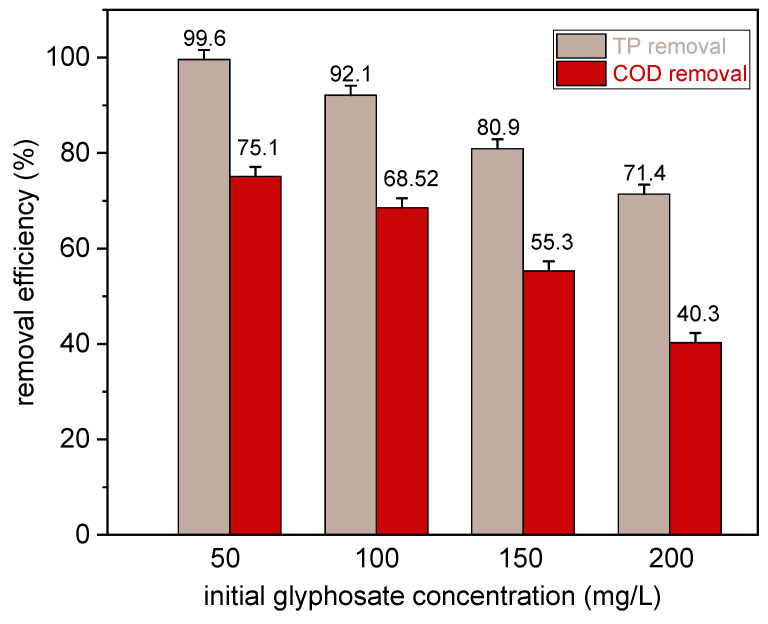
Effect of initial glyphosate concentration on TP and COD removal (Condition: pH, 3.0; the initial nCPs dosage, 0.2 g; Ca^2+^/Fe^2+^ molar ratio, 6; contact time, 75 min; RT; 150 rpm).

To assess the significance and reliability of our method, the batch degradation study was carried out with the initial glyphosate concentration ranging from 50 ppm to 200 mg/L. The efficiency of TP removal and COD removal was investigated at the initial pH = 3.0, initial nCPs dosage = 0.2 g, Ca^2+^/Fe^2+^ molar ratio = 6, contact time = 75 min and agitation speed = 150 rpm under ambient temperature. The results of total removal efficiency before and after treatment are displayed in Figure 9. It can be observed that the TP and COD removal efficiency typically reduced with the increased initial glyphosate concentration from 50 mg/L to 200 mg/L. The TP and COD removal decreased from 99.60% to 71.40% and from 75.10% to 40.30%, respectively. The lower removal efficiency at higher initial glyphosate concentration can be explained due to the greater amount of generated metabolites such as AMPA or sarcosine, which compete with glyphosate for Fenton reaction with the stationary concentration of ·OH [47,48]. Therefore, the removal efficiency is strictly restricted by the amount of free hydroxyl radicals (·OH) generated. To sum up the main points, this method was quite effective, and the initial glyphosate concentration was one of the crucial factors that had a significant impact on the glyphosate removal. As has been noted, considering implementing this process at full-scale, the strict control of operational conditions (e.g., acidic medium) and the efficiency of mineralization are worth further studying.

### 3.4. H_2_O_2_ Release

**Figure 10:**
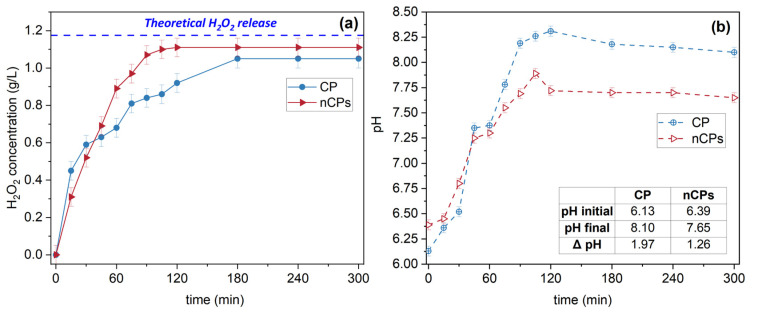
(**a**) Release of H_2_O_2_ by CP and nCPs under stirring condition (150 rpm, RT), [CaO_2_] = [nCPs] = 2.5 g/L. (**b**) The change of pH with time of CP and nCPs.

The investigation on H_2_O_2_ release by CP and nCPs was conducted in a 250 mL conical flask equipped with the agitation speed of 150 rpm at room temperature. 0.5 g of CP and nCPs was weighed out and dissolved in 200 mL distilled water before adding in the flasks, respectively. To monitor the instantaneous concentration of H_2_O_2_ generated from calcium peroxide and the instantaneous pH of solutions, 2 mL of sample was collected from each flask within 5 h. For the first 2 h, absorbance was monitored at intervals of 15 min. The results of the controlled release and the change of pH with time are displayed in Figure 10. The theoretical H_2_O_2_ release was calculated according to molecular formula. It could be observed that the rate of H_2_O_2_ released for both CP and nCPs was remarkably fast in the initial 60 min, which proved the first stage of Fenton reaction as confirmed by the previous study [44]. It is worth noting that up to 70.90% of CP and 72.20% of nCPs were converted the liquid H_2_O_2_ within the initial hour. The CP and nCPs samples continued to actively release H_2_O_2_ until the equilibrium was reached, which was expected to be around 3 h (CP) and 2 h (nCPs). Though the nCPs posed smaller particle size and larger surface area, nCPs released H_2_O_2_ relatively slowly compared to CP at the first of 30 min, due to the PEG 200 coated during the synthesis. This was because the involvement of PEG 200 functioning as a membrane on the surface of nCPs could result in a slower rate of H_2_O_2_ release [24]. The conversion percentage of nCPs (94.50%) was much higher than that of CP (89.30%) within 5 h, suggesting that nanoscale calcium peroxide performed better than conventional CP in water regarding releasing H_2_O_2_. The remainder of CP (10.70%) and nCPs (5.50%) was related to decomposition in water and oxygen generation [19]. When it came to pH changes, an increase of 1.97 for CP and 1.26 for nCPs was observed due to increased H_2_O_2_ release in the nCPs system. To conclude, nanoparticles calcium peroxide is of great efficiency as an oxidant owing to the high dispersion and smaller particle size [44].

### 3.5. Kinetic Study

**Figure 11:**
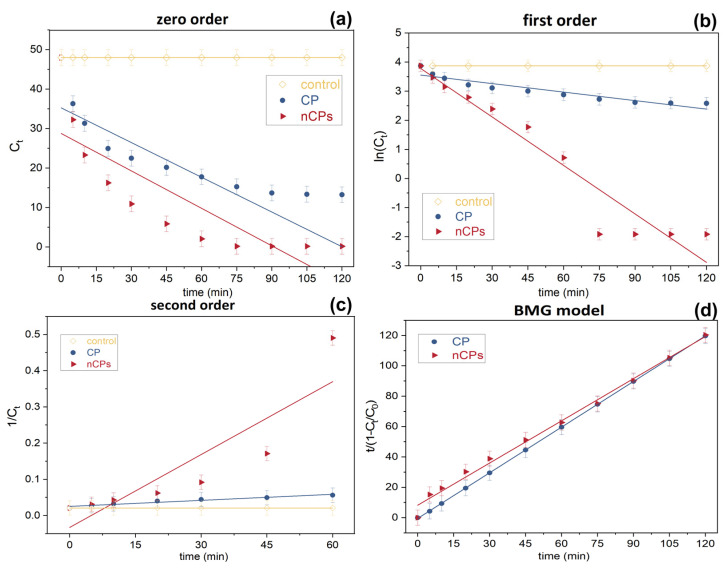
Degradation data of glyphosate using different kinetic models.

In the kinetic study, four models were utilized to study the degradation kinetic of the glyphosate based on calcium peroxide using Fenton-based system. The obtained kinetic parameters are illustrated in Figure 11 and Table 4. For second-order kinetic study, the contact time of 60 min was selected to fit with degradation data due to the feasibility of data fitting. Overall, the most appropriate model to describe glyphosate removal was the BMG model reaction kinetics (average R^2^ = 0.9965), followed by first-order kinetics (average R^2^ = 0.9336). Due to the low average correlation coefficient (average R^2^ < 0.9), the zero-order (0.7598) as well as the second order (0.8920) could not fit with the degradation data. Regarding parameter 1/m (initial degradation rate) acquired from BMG model, the initial degradation rate increased to four times in nCPs system (1/m = 0.4916), leading to the faster removal rate. That is to say, the first stage of the degradation process made the greatest contributions to the overall removal efficiency because, as was discussed before, it is a rapid stage of the Fenton reaction. By considering the parameter 1/b (maximum oxidation capacity), the same finding can be observed from the Table 4. The maximum oxidation capacity in nCPs system was 1.1799 whereas that of the CP system was only 0.9981, suggesting that the higher removal efficiency could be observed in nCPs system. When it came to the first-order kinetics model, it can be observed that the reaction rate coefficient (k_1_) in nCPs system was even six times greater than the CP system. Therefore, the reaction rate of nCPs system is considerably rapid than the CP system. The result of total removal efficiency proved this finding as well. These basic findings are consistent with previous research [13,21,35] showing that BMG model is the most appropriate model to describe the degradation data by AOPs.

### 3.6. Proposed Degradation Pathways

**Figure 12:**
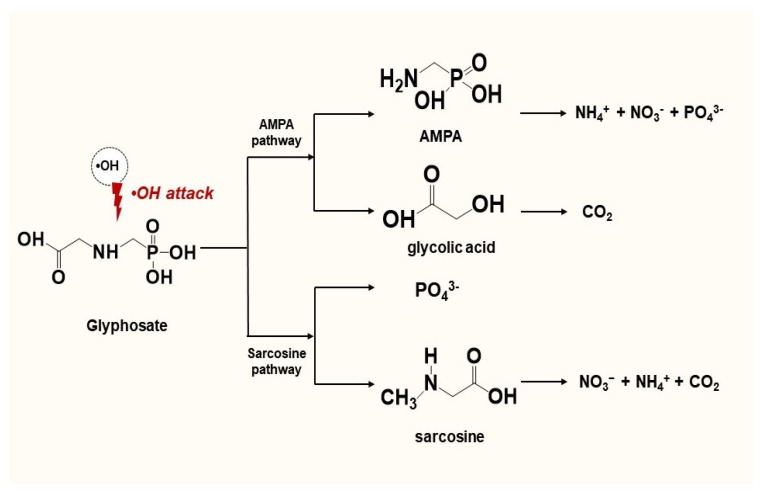
Proposed glyphosate degradation pathways via AOPs.

According to the literature [8], glyphosate degradation and mineralization by AOPs are linked to the cleavage of C-P and C-N bonds by hydroxyl radicals (·OH). Two oxidation pathways via AOPs were reported, which are AMPA pathway and sarcosine pathway [9]. Furthermore, it is common to detect these two pathways can exist separately or coexist during the degradation process [49]. The possible degradation mechanisms in the present study are displayed in Figure 12. Initially, glyphosate is attacked by hydroxyl radicals (·OH) to yield AMPA and glycolic acid (AMPA pathway), or sarcosine and PO_4_^3−^ (sarcosine pathway). Two metabolites, which are AMPA and glycolic acid, can be produced via AMPA pathway. Through the cleavage of C-P bond by means of ·OH, AMPA can be further converted to NH_4_^+^, NO_3_^−^, and PO_4_^3−^ and glycolic acid can be finally oxidized to CO_2_. On the other hand, glyphosate can be degraded into sarcosine and PO_4_^3−^ via sarcosine pathway. Subsequently, sarcosine can be further oxidized to N-contained metabolites, which can be eventually converted to NH_4_^+^, NO_3_^-^, and CO_2_. The possible mechanisms also implied that ·OH can oxidize and decompose organic matter until its total mineralization to CO_2_, H_2_O, and its corresponding inorganic salt [15,50,51]. As a result, AOPs are viewed as playing a critical role in the treatment of glyphosate containing wastewater.

## 4. Conclusions

In the present study, PEG-coated calcium peroxide nanoparticles were successfully synthesized, and the effective removal efficiency was successfully achieved. The average particle size of synthesized nCPs was approximately 40.88 nm with larger surface area of 28.09 m^2^/g and a uniform distribution. The maximum TP removal efficiency (99.60%) and total COD removal efficiency (75.10%) could be acquired at the given conditions (pH = 3.0, the initial nCPs dosage = 0.2 g, Ca^2+^/Fe^2+^ molar ratio = 6, the initial glyphosate concentration = 50 mg/L, RT). The degradation rate of glyphosate in aqueous system was rapid and equilibrium was achieved within 75 min. The degradation process of glyphosate could be most effectively described by the BMG model. The particle size and initial glyphosate concentration revealed a significant impact on glyphosate removal. Moreover, the effectiveness of the H_2_O_2_ release and the outcome of the initial glyphosate concentration study demonstrated the reliability and potential of this material. To summarize the findings of this work, calcium peroxide nanoparticles represent an efficient oxidant for glyphosate removal from aqueous systems.

## Figures and Tables

**Table 1:** The equations of kinetic equations.

Order	Equation Applied	Linear Form by Integration
Zero-order	$\frac{dC_{t}}{d_{t}}=-k_{0}$	$C_{t}=C_{0}-k_{0}\cdot t$
First-order	$\frac{dC_{t}}{d_{t}}=-k_{1}\cdot C_{t}\;$	$lnC_{t}=lnC_{0}-k_{1}\cdot t$
Second-order	$\frac{dC_{t}}{d_{t}}=-k_{2}\cdot {\left(C_{t}\right)}^{2}$	$\frac{1}{C_{t}}=\frac{1}{C_{0}}+k_{2}\cdot t$
BMG model	$\frac{C_{1}}{C_{0}}=1-\left[\frac{t}{\left(m+b\cdot t\right)}\right]$	$\frac{t}{\left[1-\left(\frac{C_{t}}{C_{0}}\right)\right]}=m+b\cdot t$

Note: *k_0_*, *k_1_*, and *k_2_* are apparent kinetic rate constants of zero-, first-, and second-order models, respectively. *t* is reaction time, and *C_t_* is the concentration at a given time *t*. where *m* and *b* are two constants concerning initial degradation rate and maximum oxidation capacity, respectively.

**Table 2:** Comparison of 2θ and d-spacing values of the standard CaO_2_ with the synthesized nCPs.

Peak Number	CaO_2_ (JCPDS-00-003-0865)			Synthesized nCPs		
	2θ (Degree)	d-Spacing (Å)	Miller Indices (h k L)	2θ (Degree)	d-Spacing (Å)	Miller Indices (h k L)
1	30.27	2.95	0 0 2	30.18	2.95	0 0 2
2	35.60	2.52	1 1 0	35.90	2.52	1 1 0
3	47.31	1.92	1 1 2	47.20	1.92	1 1 2
4	51.60	1.77	2 0 0	51.90	1.77	2 0 0
5	60.89	1.52	2 0 2	60.94	1.52	2 0 2

**Table 3:** Physicochemical properties of CaO_2_ and nCPs.

Sample	Purity (%)	Surface Area (m^2^/g)	Pore Size (nm)	Pore Volume (cm^3^/g)	Surface-Volume Ratio	Average Size (nm)	PDI
CP	65.0%	3.1079	2.365	0.3644	13.70	220.16	0.592
nCPs	75.1%	28.0860	23.1324	1.3248	21.25	40.88	0.228

**Table 4:** Comparison of kinetic models for different samples (CP and nCPs).

Sample	Removal (%)	Zero-Order		First-Order		Second-Order		BMG Model		
		k_0_	R^2^	k_1_	R^2^	k_2_	R^2^	1/m	1/b	R^2^
CP	73.53	0.2993	0.8248	0.0097	0.9385	0.0067	0.7449	0.1218	0.9981	0.9999
nCPs	99.60	0.3163	0.6949	0.0555	0.9286	5.5700	0.9411	0.4916	1.1799	0.9930
Average R^2^			0.7598		0.9336		0.8430			0.9965

## Data Availability

The data presented in this study are available on demand from the corresponding author or the first author.
